# Unseen and Undiagnosed: The Relationship of Social Isolation to Underdiagnosed Dementia

**DOI:** 10.1093/geroni/igaf122.1168

**Published:** 2025-12-31

**Authors:** Ashwin Kotwal, Irena Cenzer, Min Hee Kim, Ruijia Chen, Carla Perissinotto, Willa Brenowitz, Jacqueline Torres

**Affiliations:** University of California, San Francisco, San Francisco, California, United States; University of California, San Francisco, San Francisco, California, United States; Rutgers, New Brunswick, New Jersey, United States; Boston University School of Public Health, Boston, Massachusetts, United States; University of California, San Francisco, San Francisco, California, United States; Kaiser Permanente, Portland, Oregon, United States; University of California, San Francisco, San Francisco, California, United States

## Abstract

Social isolation may delay recognition of cognitive decline, leading to underdiagnosis or misdiagnosis of dementia. We examined the probability of undiagnosed dementia among older adults based on social isolation status using data from the U.S. Health and Retirement Study (HRS) linked to Medicare claims. Social isolation was measured through household contacts (marital status, household size, children nearby) and community engagement (volunteering, religious attendance). Probable dementia classification followed the Power-Gianattasio Expert model, maximizing accuracy across race and ethnicity subgroups, while the Bynum algorithm was applied to Medicare claims-based diagnoses. The primary outcome was dementia diagnosis status (correctly diagnosed, undiagnosed, or overdiagnosed) based on probable dementia classification in a given HRS wave and a corresponding Medicare-based diagnosis in the three years prior or one year after. We estimated associations between social isolation and dementia diagnosis status. Preliminary estimates from 2018 and 2020 indicate that 26-27% of respondents were socially isolated, 14% had probable dementia, and 8-9% had a claims-based dementia diagnosis. In 2018, socially isolated individuals had greater undiagnosed dementia than non-isolated individuals (12.7% vs. 7.3%, p < 0.01), with similar overdiagnosis rates (∼2%). In 2020, rates were similar by social isolation status (undiagnosed dementia: 9.8% vs. 7.4%; overdiagnosis: 3.7% vs. 2.7%). Social isolation may contribute to disparities in timely dementia diagnosis due to reduced access to care and fewer social contacts recognizing cognitive changes. These findings suggest claims-based research may underestimate dementia cases among isolated individuals. Addressing social isolation as a public health concern may help mitigate disparities in dementia detection and care.

